# The Abundance of Pink-Pigmented Facultative Methylotrophs in the Root Zone of Plant Species in Invaded Coastal Sage Scrub Habitat

**DOI:** 10.1371/journal.pone.0031026

**Published:** 2012-02-24

**Authors:** Irina C. Irvine, Christy A. Brigham, Katharine N. Suding, Jennifer B. H. Martiny

**Affiliations:** 1 Department of Ecology and Evolutionary Biology, University of California Irvine, Irvine, California, United States of America; 2 Santa Monica Mountains National Recreation Area, United States National Park Service, Thousand Oaks, California, United States of America; 3 Department of Environmental Science, Policy and Management, University of California, Berkeley, California, United States of America; J. Craig Venter Institute, United States of America

## Abstract

Pink-pigmented facultative methylotrophic bacteria (PPFMs) are associated with the roots, leaves and seeds of most terrestrial plants and utilize volatile C_1_ compounds such as methanol generated by growing plants during cell division. PPFMs have been well studied in agricultural systems due to their importance in crop seed germination, yield, pathogen resistance and drought stress tolerance. In contrast, little is known about the PPFM abundance and diversity in natural ecosystems, let alone their interactions with non-crop species. Here we surveyed PPFM abundance in the root zone soil of 5 native and 5 invasive plant species along ten invasion gradients in Southern California coastal sage scrub habitat. PPFMs were present in every soil sample and ranged in abundance from 10^2^ to 10^5^ CFU/g dry soil. This abundance varied significantly among plant species. PPFM abundance was 50% higher in the root zones of annual or biennial species (many invasives) than perennial species (all natives). Further, PPFM abundance appears to be influenced by the plant community beyond the root zone; pure stands of either native or invasive species had 50% more PPFMs than mixed species stands. In sum, PPFM abundance in the root zone of coastal sage scrub plants is influenced by both the immediate and surrounding plant communities. The results also suggest that PPFMs are a good target for future work on plant-microorganism feedbacks in natural ecosystems.

## Introduction

Methylotrophic bacteria utilize single carbon (C_1_) compounds for energy and assimilation and are an important component of the global carbon cycle [1], [2]. One group of methylotrophs, pink-pigmented facultative methylotrophic bacteria (PPFMs), is distinguished based on their formation of pink to red colonies on selective isolation media. Classified within the genus *Methylobacterium*, PPFMs are facultative methylotrophs, using both single and multicarbon compounds.

PPFMs are associated with the roots, leaves and seeds of most terrestrial plants, and many are thought to be phytosymbionts [3]. The bacteria use C_1_ compounds, such as methanol, generated by growing plants during cell division. In return, they can positively affect plant growth and survival [3]. Much of the evidence for these positive effects derives from agricultural systems, where PPFMs have been shown to improve seed germination, crop yield, pathogen resistance and drought stress tolerance [4], [5], [6].

There are at least two mechanisms by which PPFMs can positively affect plants, particularly in dry climates. First, PPFMs excrete auxins and cytokinins, plant growth hormones that influence germination and root growth and play critical roles in a plant's response to water stress [7], [8]. In dry conditions, plants that send their roots deep quickly after germination may gain a competitive advantage over more shallowly rooted species. Second, PPFMs exude osmoprotectants (sugars and alcohols) on the surface of host plants [3]. This matrix may help protect the plants from desiccation and high temperatures.

In a recent study, we investigated the effect of PPFMs on several coastal sage scrub (CSS) plant species [9]. CSS is a low shrubland community that once dominated the Mediterranean-type climate regions of coastal California. Most native CSS plants are perennial, drought-deciduous species that can persist during dry, hot (+33°C) summers. Invasive species, many of which are annuals, have significantly fragmented and diminished the quality of CSS habitat [10]. In a combination of laboratory and field experiments, we found that PPFM or methanol addition stimulated germination and/or growth of two native species (*Artemisia californica* and *Nasella pulchra*), but not that of three invasive species [9]. These results suggest that PPFMs may have a greater benefit to native than invasive CSS species.

Given these interactions, we aimed to better understand the distribution of PPFMs in the CSS habitat, particularly in the root zones of native and invasive plant species. Despite their potential importance for plant growth and survival, most of what is known about PPFM distribution and diversity comes from agricultural settings. PPFMs and more broadly, *Methylobacterium* species, often make up the majority of cultivable heterotrophic bacteria in the phyllosphere (on leaf surfaces) of many plant families [11], [12], [6], [13], [14]. Several studies report that PPFM densities on leaves differ among crop species [15], [12], [16], suggesting that plant species vary in the quality of habitat that they provide for PPFMs. In addition, PPFM abundance and composition also appears to vary in the rhizosphere (next to the roots) of various crop species [13], [16], [17]. In natural ecosystems, PPFMs have been detected on the phyllosphere of temperate and tropical plant species worldwide [18], [19]. However, few studies have shown that methylobacteria or PPFM abundance varies among plant species in these ecosystems [18], [20], [21]. Further, we are unaware of any studies that investigate the abundance or composition of PPFMs in the rhizosphere or soils of natural terrestrial ecosystems.

Here we surveyed PPFM abundance from the root zones of a variety of 10 common native and invasive species in CSS. We addressed three questions: (1) Does PPFM abundance in the root zone differ by CSS plant species? (2) If so, does this abundance vary by native or invasive plant species? (3) Is PPFM abundance affected by the surrounding plant community (other plant species in the area)?

We estimated PPFM abundance from plant root zone soil using the most probable number (MPN) technique. Because PPFMs are defined by their appearance on a selective media, quantification by culturing is appropriate for this group. We predicted that PPFM root zone abundance would vary among plant species in natural systems, as observed in crop species. Further, we expected that PPFM abundance under native plant species would be higher than those under invasive plant species, hypothesizing that native plants adapted to the CSS habitat would be associated with more PPFMs to mitigate drought and heat stress. Finally, given the local scale of potential plant-PPFM interactions, we expected that root zone abundance would be determined by the immediate plant species and not the surrounding community (other native or invasive plant species).

## Materials and Methods

### Study site description and sample collection

The study was conducted in the Santa Monica Mountains National Recreation Area (SMMNRA, U.S. National Park Service) in southern California, USA. The Santa Monica Mountains have a Mediterranean-type climate with most precipitation in the cool winter months (December through February, rainfall typical ranges between 250–330 mm/yr) followed by long, hot and dry conditions for the remainder of the year. The SMMNRA is bounded by the heavily populated Los Angeles and Ventura counties (pop. ∼10 million and ∼750,000 respectively). Invasive species threaten critical habitat to about 100 sensitive, threatened or endangered plant and animal species in the park.

In early June 2007, we identified 10 plant invasion gradients (or “sites”) in CSS within a 0.5 km^2^ area of the Satwiwa/Rancho Sierra Vista region of the SMMNRA. We laid one transect (10–32 m in length) through each invasion gradient, such that one end of the transect fell in a stand of all native plant species and the other end in a stand of all invasive species. Every effort was made to select sites with qualitatively similar soil type (clay), soil moisture (dry), slope (zero to 5 degrees), and aspect (zero to north or northwest). The transects included native species that are common constituents of CSS assemblages within the SMMNRA: *Artemisia californica* (California sagebrush); *Eriogonum fasciculatum* (California buckwheat); *Nassella lepida* (foothill needle grass); *Nassella pulchra* (purple needle grass); and *Baccharis pilularis* (coyote brush). Invasive species in the transects included common, problematic invaders that are subject to ongoing control efforts at the SMMNRA: *Carduus pycnocephalus* (Italian thistle); *Conium maculatum* (poison hemlock); *Foeniculum vulgare* (fennel); *Hirschfeldia incana* (shortpod mustard); and *Phalaris aquatica* (Harding grass). Not all species occurred at each site, although there was overlap between sites.

We established five, ∼1 m^2^ plots along each of the 10 transects. One plot was placed at the “pure” native (0% invaded) end of the transect, and one plot was placed at the “pure” invasive (100% invaded) end. Three additional plots were placed in “mixed” stands along the transect, such that the plots contained approximately 20, 50, and 80% invasive plant cover as determined by eye.

We then sampled soil from the root zones (∼100 g, 5 cm deep at the base of the root crown) of green (photosynthesizing) plants from the plots. We took samples from 3 individuals of each plant species in each plot. Thus, the number of samples from a plot depended on the number of species present in a plot. Further, not all species present at a site (Table 1) were present in all the mixed plots. In total, we collected 246 soil samples, each of ∼100 g. As typical for early June, soils were dry at the time of collection. Samples were stored at 4°C at the University of California, Irvine for 60 days and processed in random order over a two week period.

**Table 1:** PPFM abundance associated with the root zone of the different plant species.

Plant Species	Life History and Form	Native CSS or Invasive	Site	Mean CFU/g dry soil ± SEM
*Artemisia californica*	PS	Native	1,4,5,7,8	5595±1620
*Baccharis pilularis*	PS	Native	7,10	5809±2146
*Eriogonum fasciculatum*	PS	Native	2	7243±2916
*Nassella lepida*	PB	Native	2,9	12774±9753
*Nassella pulchra*	PB	Native	1,3,6	5564±1516
*Carduus pycnocephalus*	AH	Invasive	1,2,8	15282±4076
*Conium maculatum*	BH	Invasive	8	12743±7818
*Foeniculum vulgare*	PH	Invasive	3,5	3882±576
*Hirschfeldia incana*	AH	Invasive	6,7,8,10	12012±2941
*Phalaris aquatica*	PB	Invasive	4,9	2817±470

AH = annual herb, BH = biennial herb, PB = perennial bunchgrass, PH = perennial herb, PS = perennial shrub. Mean MPN ± SEM is the average across all sites where the plant species was found (3 samples/species at each zone of invasion both pure and mixed, N = 246).

### Most Probable Number (MPN) Dilutions

In the lab, each soil sample was homogenized, sieved and divided into three sub-samples prior to serial dilution. To estimate PPFM abundance, a ten-fold dilution series (1∶10 to 1∶100,000) for each sub-sample was prepared using sterile, modified nitrate mineral salts (NMS) medium [22] with methanol (1.0% vol/vol) as the sole carbon source in the media. Cycloheximide (100 mg/L) was added to the media to prevent fungal growth. Homogenized soil (2 g) was suspended directly in NMS medium and then further diluted. The dilutions were plated in triplicate into 1.5 mL/well uncoated, sterile microtiter plates. The plates were covered and incubated at 30°C for 14 days. Wells with pink growth at 14 days were scored as positive for PPFMs.

### PCR and Sequencing

To confirm that the pink bacteria in the MPN dilutions were members of the genus *Methylobacterium*, we performed polymerase chain reaction (PCR) amplification and sequencing on nine purified colonies. For each colony, we targeted the methanol dehydrogenase gene (*mxa*F) and the 16 S small sub-unit ribosomal DNA (16 S SSU). The *mxa*F gene was selected for amplification because it has been found in all *Methylobacterium* to date [23]. Nine positive wells from the MPN plates were chosen randomly and streaked on NMS agar (methanol 1.0% vol/vol, 100 mg cycloheximide/L media) and incubated as above. A colony from these plates was re-streaked for further purification. One colony from each of the second streak plates was transferred to NMS broth and incubated until log phase (30°C, shaken at 150 rpm for 7 days). Total genomic DNA was extracted (Promega Wizard® Genomic DNA Purification Kit) from each of the pure cultures. Primers used to target the *mxa*F gene (*mxa*F1003 forward and *mxa*F1541 reverse [24]) yielded a 538 bp amplicon. We also used universal eubacterial primers to target the highly conserved 16 S small sub-unit ribosomal DNA (pA forward & pH'reverse, [25]) yielding a 1500 bp amplicon. PCR conditions for the *mxa*F gene were as follows: (25-µl reaction vol.) initial denaturing 95°C (5 min.), anneal at 55.6°C (30 sec.), extension at 72°C (40 sec.), 30 cycles total with a final extension step of 72°C (5 min). [Final concentration: forward and reverse primers 250 nM, 1 unit Taq polymerase, MasterAmp™ 1× Premix F (Epicenter Biotechnologies - Wisconsin, USA)]. PCR conditions for the 16 S SSU rDNA gene were: initial denaturing 95°C (4 min), anneal at 55°C (40 sec), extension at 72°C (2 min) for 30 cycles with a final extension step of 72°C (5 min). [Final concentration: forward and reverse primers 125 nM, 1 unit Taq polymerase, MasterAmp™ 1× Premix F (as above)]. PCR products were visualized by gel electrophoresis and the target bands were excised and purified following the manufacturer's protocol (MinElute® Gel Extraction Kit - Qiagen Sciences, Maryland, USA) prior to sequencing with the forward primer (Agencourt - Massachusetts, USA). Sequences were compared to GenBank accessioned sequences using the blastn algorithm. The sequences have been submitted to GenBank under the accession numbers HQ219729-HQ219746.

### Statistical Analysis

To capture variation in PPFM abundance across different sites, we sampled from a variety of locations. This sampling pattern also meant that the plant species did not occur at every site, such that we are not able to test for interactive effects of site and plant species on PPFM abundance. Thus, we first tested for overall site differences in PPFM abundance with a one-way ANOVA. For subsequent analyses, we combined the data from all sites. Additionally, we tested for differences in PPFM abundances among the three mixed stand levels (20, 50 & 80% invaded). Since there were no significant differences among these levels, we combined these samples into one “mixed” category for all further tests. We then performed a two-way ANOVA to test for differences in PPFM abundance by plant species and by the surrounding plant community (i.e., whether in a pure or mixed stand). Because there was no significant interaction between species and pure/mixed stands, we could then use a one-way ANOVA to test whether PPFM abundance varied among native vs. invasive (or alternatively, annual/biennial vs. perennial life history). For all analyses, we ln-transformed the estimates of PPFM abundance to improve normality for the statistical tests. We performed the tests using the JMP 7.02 software (SAS Institute, Inc.). We present the back-transformed means+one standard error of the mean in the figures.

## Results

### PCR and Sequencing

As expected, the pink bacteria that we cultured all appeared to belong to the genus *Methylobacterium*. The *mxa*F sequences of the isolates were most similar to *Methylobacterium*. In particular, 2 of 9 isolates had a ≥99.2% pairwise similarity to *Methylobacterium extorquens* and 5 of 9 had a ≥98.8% similarity to the cultured *Methylobacterium* sp. F3.2 strain. The remaining sequences were most similar to *M. dichloromethanicum* and *M. rhodinium* (99.4%). The 16 S sequences yielded similar results; 4 of 9 sequences were most similar (≥99.2%) to *M. extorquens* and the remaining most similar (≥99.3%) to three cultured *Methylobacterium* species strains (Table 2).

**Table 2:** Comparison of most similar cultured isolate to GenBank Sequences (blastn) for 16 SSU ribosomal DNA and the methanol dehydrogenase gene, *mxa*F.

	*mxa*F			16 S rDNA		
Isolate	Accession #	Description	% Pairwise Identity	Accession #	Description	% Pairwise Identity
1	FJ157958	*Methylobacterium* sp. F3.2	98.8	FJ157961	*Methylobacterium* sp. 1b.3	99.3
8	FJ157958	*Methylobacterium* sp. F3.2	98.8	AM910536	*Methylobacterium sp.* F38	100.0
9	GU353343	*Methylobacterium* sp. F3.2	98.8	CP001510	*M. extorquens* AM1	99.7
4	GU353343	*Methylobacterium* sp. F3.2	98.8	AM910536	*Methylobacterium* sp. F38	99.5
6	GU353343	*Methylobacterium* sp. F3.2	98.8	FJ157976	*Methylobacterium* sp. JT1	99.4
2	U70527	*M. rhodinum*	99.4	CP000908	*M. extorquens* PA1	99.2
3	EF562465	*M. dichloromethanicum* KACC 11438	99.4	AF531770	*M. extorquens*	99.5
7	AJ878068	*M. extorquens* DM4	99.2	AB175632	*M. extorquens*	99.6
10	AB455974	*M. extorquens* NRIC 0601	99.6	FJ157961	*Methylobacterium* sp. 1b.3	99.3

### PPFM Abundance

PPFMs were present in every soil sample, from a minimum of 10^2^ to a maximum of 10^5^ CFU/g dry soil. There was no obvious fungal or non-target bacterial growth observed in the MPN cultures. PPFM abundance in the root zones varied significantly among sites (P = 0.0017); however, due to the changing identity of plant species present at each site, we cannot determine whether these site differences were due to the particular plant species present or abiotic effects. To examine this question further, we tested whether PPFM abundance differed in the pure stands of the four species that occurred at more than two sites (*A. californica*, *N. pulchra*, *C. pycnocephalus*, *H. incana*). Abundance did not differ across sites for any of the species (one-way ANOVA; P = 0.09–0.31), suggesting that plant species is more important to PPFM abundance than direct abiotic effects.

**Figure 1:**
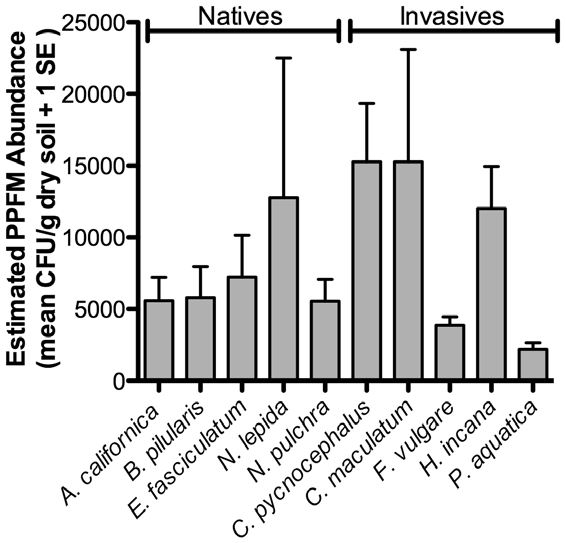
Average PPFM abundance for each species (pure and mixed stands pooled). Two-way ANOVA, Factor: species (F_1,9_ = 3.15, P = 0.0034, N = 82). Species with the same letters are not significantly different from each other (Tukey post hoc test α = 0.05, Q = 3.64). Error bars were constructed with one standard error of the mean for all soil samples taken under each plant species.

Overall, PPFM abundance differed significantly among plant species (P = 0.0034; Table 1, Figure 1). The natives, *E. fasciculatum*, *B. pilularis*, *A. californica* and *N. pulchra* showed mean PPFM abundances ranging from 5,600–7,200 CFU/g dry soil. The highest mean PPFM abundance was found under the invasive thistle, *C. pycnocephalus* (1.5×10^4^ CFU/g dry soil), followed by the native *N. lepida* and invasives *C. maculatum* and *H. incana*.

**Figure 2:**
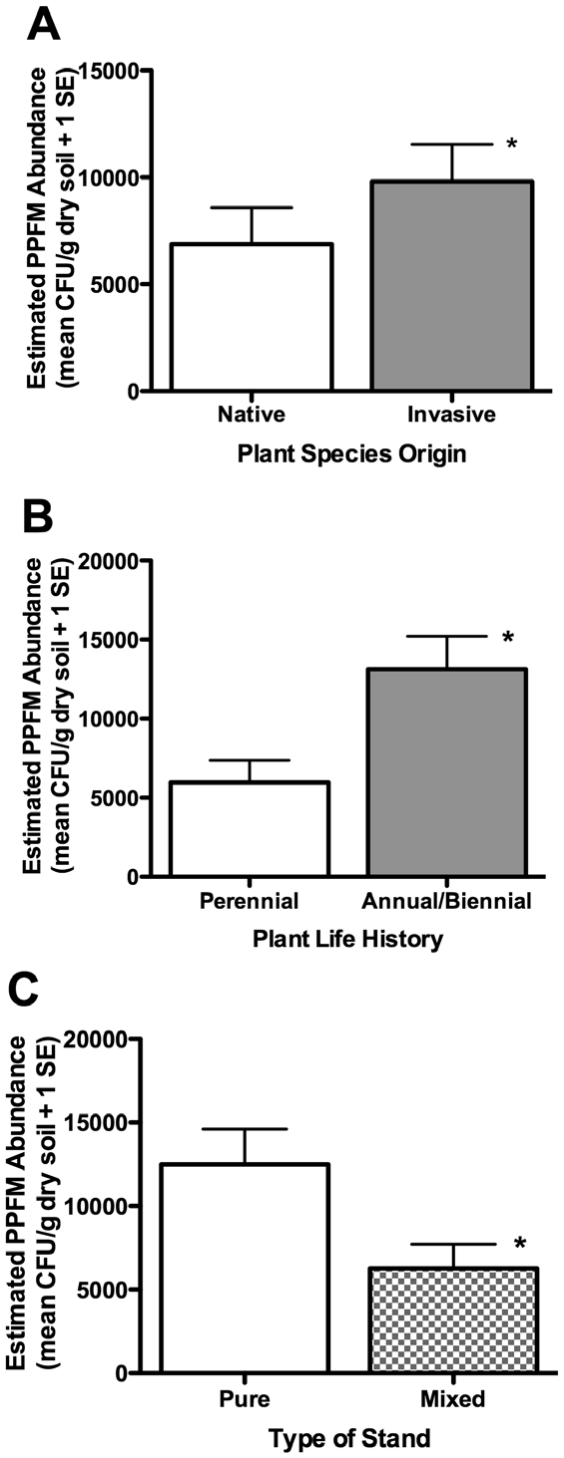
Average PPFM abundance in the root zone by species origin, life history, and pure and mixed stands. (a) Native versus invasive species: one-way ANOVA, (mixed and pure stands pooled) F_1,80_ = 5.21, P = 0.0251, N = 82; (b) Annual/biennial versus perennial life history: one-way ANOVA, (mixed and pure stands pooled) F_1,80_ = 20.45, P<0.0001, N = 82; (c) Pure versus mixed stands: two-way ANOVA, F_1,9_ = 4.06, P = 0.048, N = 82. Error bars were constructed with one standard error of the mean.

The lowest mean PPFM abundance occurred under the two perennial invasives, *P. aquatica* and *F. vulgare*, 2.8–3.9×10^3^ CFU/g dry soil, respectively. PPFM abundance was 1.4 times higher in the root zones of invasive plant species than native plant species (9,808±1,733 SEM versus 6,878±1,715 SEM, respectively; P = 0.025; Figure 2a). To examine whether this difference might be related to plant life history, we classified the species as annual/biennial or perennial and performed the same analysis. Indeed, annual/biennials had over 2 times more PPFMs than perennials (13,130±2,251 SEM versus 5,963±1,367 SEM, respectively; P<0.0001; Figure 2b).

Finally, the number of PPFMs in the root zone of a particular species appeared to be affected by the broader, surrounding plant community. We found 50% fewer PPFMs in the mixed species stands than in pure stands (6,262±809 SEM versus 12,487±3342 SEM, respectively; P = 0.0476; Figure 2c). There was no interactive effect of the focal plant species and the type (mixed/pure) of community stand on PPFM abundance (P = 0.553).

## Discussion

PPFMs were ubiquitous among our soil samples from plant root zones in coastal sage scrub habitat. As far as we are aware, this study is first to demonstrate that PPFM abundance in the root zone varies among plant species in a natural ecosystem. This variation could be due in part to differences in root growth habitats among the plant species or in the amount and types of carbon compounds exuded from them. The result parallels prior work that shows that phyllosphere PPFM abundance varies among plant species in natural ecosystems [21], [18].

Contrary to our original hypothesis, native plant species generally had significantly fewer PPFMs than invasives. However, the pattern observed may be driven primarily by plant life history. All of the native species in this study were slow growing, drought-deciduous perennials. There were no native annual species in the transects (native annuals have a patchy distribution in CSS at SMMNRA), whereas three of the five invasive species were annuals. Supporting this interpretation, the two perennial invasives had the fewest PPFMs of all of the plants. Overall, annual/biennial species had twice as many PPFMs in their root zones than the perennial species. Perhaps drought deciduous perennials may not be as favorable to PPFMs because of their slower growth compared to annuals [15], [26], [16]. PPFMs consume the C_1_ by-products of cell division, thus faster plant growth may result in faster PPFM growth and higher abundances. In future studies, the relative importance of native/invasive status versus life history on PPFM abundance should be tested in a system with better representation of the species' types.

The fact that we found on average 50% fewer PPFMs in the mixed stands (native and invasive) versus pure stands (only natives or only invasives) suggests that the surrounding plant community influences PPFM abundance in another plant's root zone. Many studies have found that plants exude complex organics and extracellular enzymes from their roots to compete for space and resources [27], [28]. Some of these compounds have antimicrobial properties that could be affecting PPFMs [29]. Further, there is evidence that the amount and/or potency of phytotoxins released by invasive plant species changes when in competition with native species [30], [31]. Thus, the reduction in PPFM abundance in mixed stands relative to pure stands could be due to the antimicrobial properties of phytotoxins.

Much work remains to be done to identify the particular environmental and biotic factors influencing PPFM abundance. For instance, the site level effects on PPFM abundance that we found are presumably due to plant composition, but this is affected by abiotic conditions. Though we attempted to minimize the abiotic conditions between sites (e.g., slope, aspect, soil type and soil moisture), minor differences in these variables (or other uncontrolled variables) may also be driving some of the variation in PPFM abundance. Further, the sampling design here did not allow us to test for the effect of plant species by site interactions on PPFM abundance. In addition, PPFM abundance is known to vary over the growing season [15]; therefore, it would be useful to compare these results (from late spring) to other times of year, such as during the plants' peak growing season (in the winter/spring) or when the perennial species are dormant (in the late summer/fall). Finally, given the effect of the surrounding plant composition on root zone PPFMs, it may also be important to consider the effect of the surrounding plant density.

The differential distribution of PPFMs among CSS plant species provides further motivation for controlled experimental studies on PPFMs in non-crop species. In particular, further work should examine how the genetic composition of PPFMs varies in natural ecosystems. In addition, experiments in CSS (e.g., Irvine et al. 2011) and other habitats are needed to test whether the relative strength of PPFM-plant interactions varies among native and invasive plant species. If so, PPFMs could play a role in structuring plant communities generally [32]. Plants and soil microorganisms are also known to alter the success of invasive plants into native communities; however, these studies have focused on nitrogen-fixers, mycorrhizal fungi, and soil pathogens [33], [34], [35], [36]. PPFMs might offer a promising direction for future investigations of plant-microorganism feedbacks and native plant community restoration.
